# Unlocking the Hidden Potential of Rosemary (*Salvia rosmarinus* Spenn.): New Insights into Phenolics, Terpenes, and Antioxidants of Mediterranean Cultivars

**DOI:** 10.3390/plants13233395

**Published:** 2024-12-03

**Authors:** Andrea Baptista, Felicia Menicucci, Cecilia Brunetti, Luana Beatriz dos Santos Nascimento, Dalila Pasquini, Francesca Alderotti, Cassandra Detti, Francesco Ferrini, Antonella Gori

**Affiliations:** 1 Institute for Sustainable Plant Protection, National Research Council of Italy (CNR), Via Madonna del Piano 10, Sesto F.no, I-50019 Florence, Italy; andreacarolina.baptistaruiz@unifi.it (A.B.); felicia.menicucci@ipsp.cnr.it (F.M.); francesca.alderotti@ipsp.cnr.it (F.A.); francesco.ferrini@unifi.it (F.F.); antonella.gori@unifi.it (A.G.); 2 Department of Agriculture, Food, Environment and Forestry, University of Florence, Viale delle Idee 30, Sesto F.no, I-50019 Florence, Italy; luananascimento@ufrj.br (L.B.d.S.N.); dalila.pasquini@unifi.it (D.P.); cassandra.detti@unifi.it (C.D.)

**Keywords:** antioxidant activity, polyphenols, phytochemicals, terpenes, *Salvia rosmarinus* Spenn.

## Abstract

Rosemary (*Salvia rosmarinus* Spenn. syn. *Rosmarinus officinalis* L.) is a Mediterranean aromatic species used both as an official herb and as a spice. Different cultivars may exhibit diverse phytochemical compositions, making a comprehensive chemical characterization pivotal for a targeted selection of valuable cultivars. This study aimed to characterize and compare the phenolic and terpene composition and content of leaf extracts of six Mediterranean rosemary cultivars: ‘Alba’, ‘Arp’ ‘Ginger’, ‘Gorizia’, ‘Tuscan Blue’, and ‘Roseus’. HPLC-DAD analysis revealed a similar phenolic composition in all the cultivars, but quantitative differences were observed. The main compounds were carnosic acid derivatives, flavonoids (e.g., luteolin, apigenin, and quercetin glucosides), rosmarinic acid, caffeic acid, and other hydroxycinnamic acid derivatives. The highest phenolic content was found in ‘Alba’, with a predominance of carnosic acid derivatives, whereas the lowest was found in ‘Ginger’ and ‘Gorizia’. The GC-MS analysis evidenced quantitative differences among the cultivars. Particularly, ‘Alba’ contained the highest terpene content, whereas ‘Arp’ and ‘Gorizia’ showed the lowest values. Regarding the antioxidant activity, ‘Alba’ exhibited the highest values as regards phenols, while for terpenes, the highest ones were obtained for ‘Ginger’ and ‘Tuscan Blue’. Significant Pearson correlations were obtained between the total phenol/terpene content and the antioxidant activity. The chemical characterization of these cultivars provides relevant information to produce the rosemary phytocomplexes, finding multiple industrial applications.

## 1. Introduction

Rosemary (*Salvia rosmarinus* Spenn., syn. *Rosmarinus officinalis* L.) is an aromatic evergreen shrub distributed throughout the Mediterranean region that exhibits a variety of growth habits, morphological traits, flower colors, and aromatic features [1]. This evergreen perennial shrub can grow up to 2 m tall and has aromatic, leathery, and linear leaves. These are dark green on the upper side and gray on the underside, measuring 1.0–2.5 cm in length and 1–3 mm in thickness [2]. Rosemary flowers are small, with colors ranging from light blue to lilac [2]. The strong fragrance of the leaves and flowers is attributed to the volatile oils stored in specialized glandular trichomes [3].

Traditionally added to foods as a spice, rosemary represents a natural source of high-value phytochemicals, finding numerous applications in various fields [4,5,6], such as the food, cosmetic, and pharmaceutical industries [7,8,9]. Very recent phylogenetic studies have led to the fusion of the *Rosmarinus* and *Salvia* genera. Therefore *S. rosmarinus* Spenn. is now the correct name of the species, while *R. officinalis* and its cultivars and subspecies are accepted synonyms that are still widely and traditionally used [8,10].

Plants synthesize a vast array of secondary metabolites that play crucial roles in ecological interactions and defense mechanisms [11]. The main classes of plant secondary metabolites include terpenoids, phenolic compounds, alkaloids, and glucosinolates [12]. There is a growing interest in these compounds, finding multiple applications as antimicrobial, antifungal, anticarcinogenic, antiviral, anti-mutagenic, and antioxidant agents [5,13,14]. For example, the anti-inflammatory and anticarcinogenic properties of rosemary are primarily attributed to its high concentration of phenolics, such as phenolic diterpenes, including carnosic acid, carnosol, and rosmarinic acid [10,11,12,13]. Additionally, terpenoids and flavonoids present in rosemary have been shown to possess significant antibacterial and antiviral effects [6].

In rosemary, polyphenols and terpenes are primarily located in the stems, flowers, and leaves [15]. The concentration of these metabolites varies considerably depending on the plant tissue, age, cultivation conditions, time of harvest, and exposure to biotic and abiotic factors [4,16]. Differences in the composition and concentration of secondary metabolites are also observed among different varieties and cultivars of the same plant species. These variations may be influenced by inherent genetic factors or by the transcription of different genes triggered by environmental conditions, cultivation practices, or developmental stages of the plants. As a result, the derived products, such as plant extracts, essential oils, and other phytochemicals, often exhibit substantial variability in their chemical profiles and biological activities. This variability can affect the efficacy, strength, and quality of these products, which is important for their applications in food, medicine, cosmetics, and other industries. Therefore, understanding these differences is crucial for standardizing plant-based products and ensuring their consistent performance across various applications. For instance, the analysis of the essential oils extracted from two rosemary varieties (*var. typicus* and *var. troglodytorum*) revealed a different chemical composition, which was correlated with the differing antimicrobial and antioxidant activities exhibited by the two essential oils [17]. Additionally, it is possible to select specific cultivars of rosemary based on the variability in their secondary metabolite composition. For example, cultivars with high camphor content exhibit excellent performance in applications such as anti-inflammatory therapeutics and respiratory therapies [18,19]. Likewise, the high content of phenolic acids and diterpene phenols, such as rosmarinic acid, carnosic acid, ursolic acid, and carnosol, found in rosemary, have beneficial effects on the pathogenesis of respiratory diseases [18,20,21]. Polyphenols, phenolic diterpenes, and terpenes are very appealing also for the food industry, as antioxidants and flavoring additives of many products. For instance, carnosic acid has been reported to exhibit higher antioxidant properties compared to carnosol, and cultivars with a high content of this diterpene demonstrated optimal performance as food antioxidants [22,23,24,25].

Determining the chemical composition of different rosemary cultivars, particularly concerning polyphenols, phenolic diterpenes, and terpenes, is crucial to address the need of the pharmaceutical, nutraceutical, and cosmetic industries making an extensive use of this species. Indeed, extracts obtained from dried rosemary leaves serve as food preservatives to inhibit oxidation, in accordance with food additive E-392 [24]. This study aims to provide a comprehensive chemical characterization of rosemary extracts obtained from six *S. rosmarinus* cultivars: ‘Albus’ (or ‘Alba’), ‘Gorizia’, ‘Roseus’, ‘Tuscan Blue’, ‘Arp’, and ‘Ginger’ (or ‘Green Ginger’). The cultivars used in this study were grown under the same conditions (on-demand irrigation and natural lighting) and were sampled at the same time, thus allowing comparison of their composition and antioxidant activities. The objective is to discriminate the six cultivars based on their polyphenolic and terpene composition, the content of the different classes of secondary metabolites as well as their relationships with antioxidant activity, assessed using the Ferric Reducing Antioxidant Power (FRAP) and 2,2-diphenyl-1-picrylhydrazyl (DPPH) assays.

## 2. Results

### 2.1. Analysis of Phenolic Compounds in Rosemary Cultivars

**Figure 1:**
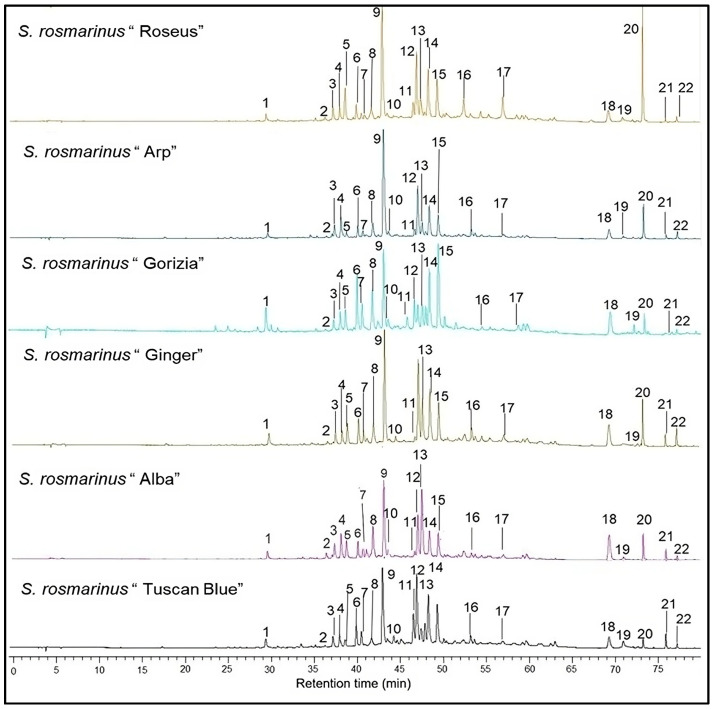
Representative HPLC-DAD chromatograms (280 nm) of the phenolic and phenolic diterpenes extracts of the six rosemary (*Salvia rosmarinus* Spenn.*)* cultivars: ‘Alba’, ‘Arp’, ‘Gorizia’, ‘Ginger’, ‘Roseus’, and ‘Tuscan Blue’. The number of peaks corresponds to the following sequence: **1**—Caffeic acid; **2**—Caffeic acid derivative; **3**—Quercetin derivative 1; **4**—Quercetin derivative 2; **5**—Luteolin derivative 1; **6**—Quercentin derivative 3; **7**—Hesperidin; **8**—Apigenin derivative 1; **9**—Rosmarinic acid; **10**—Luteolin derivative 2; **11**—Apigenin derivative 2; **12**—Luteolin derivative 3; **13**—Luteolin derivative 4; **14**—Apigenin derivative 3; **15**—Luteolin derivative 5; **16**—Luteolin derivative 6; **17**—Luteolin derivative 7; **18**—Rosmarinic acid derivative; **19**—Carnosol; **20**—Carnosic acid; **21**—Carnosic acid derivative 1; **22**—Carnosic acid derivative 2.

Twenty polyphenols and phenolic diterpenes (canosol, carnosic acid and its derivatives) were identified and quantified in the extracts of all the rosemary cultivars (Figure 1) using a high-performance liquid chromatography-diode array detection (HPLC-DAD) analysis. Qualitatively, all the extracts were very similar, with the same peaks detected in all the samples (see Figure 1), varying mainly in quantity.

The different compounds were grouped into three main classes based on their structural similarities. The first class is that of the carnosic acid derivatives, and includes carnosol, carnosic acid, and carnosid acid derivatives. The second class is that of flavonoids, encompassing the following compounds: quercetin and its derivatives, luteolin and its derivatives, apigenin and three of its derivatives, and hesperidin. The third class is that of the hydroxycinnamic acid derivatives and includes caffeic acid and its derivatives, among which rosmarinic acid is the most abundant one (Table 1 and Table S1).

Statistical analyses showed significant differences (*p* < 0.05) in the mean total phenolic content among the six cultivars, as shown in Table 1. The highest concentration of phenolics was detected in ‘Alba’ (30.99 ± 4.45 mg/g DW), while all the other cultivars showed similar levels of total phenols (Table 1).

The results of the quantitative analysis of the different classes of compounds within each cultivar indicated that phenolic diterpenes were the most represented in all the cultivars. For phenolic diterpenes, the highest content was found in ‘Alba’ (24.85 ± 2.72 mg/g DW), whereas the highest flavonoid content was recorded in ‘Tuscan Blue’ (6.36 ±1.43 mg/g DW), followed by ‘Alba’ (5.32 ± 1.51 mg/g DW), and ‘Arp’ (4.98 ± 1.07 mg/g DW), which did not statistically differ. The ratio between carnosic acids and carnosol, reported accordingly with Mira-Sánchez et al., 2020 [24], showed optimal values in ‘Gorizia’, ‘Alba’ and ‘Arp’ (Table 1).

### 2.2. Analysis of Terpenes in Rosemary Cultivars

**Figure 2:**
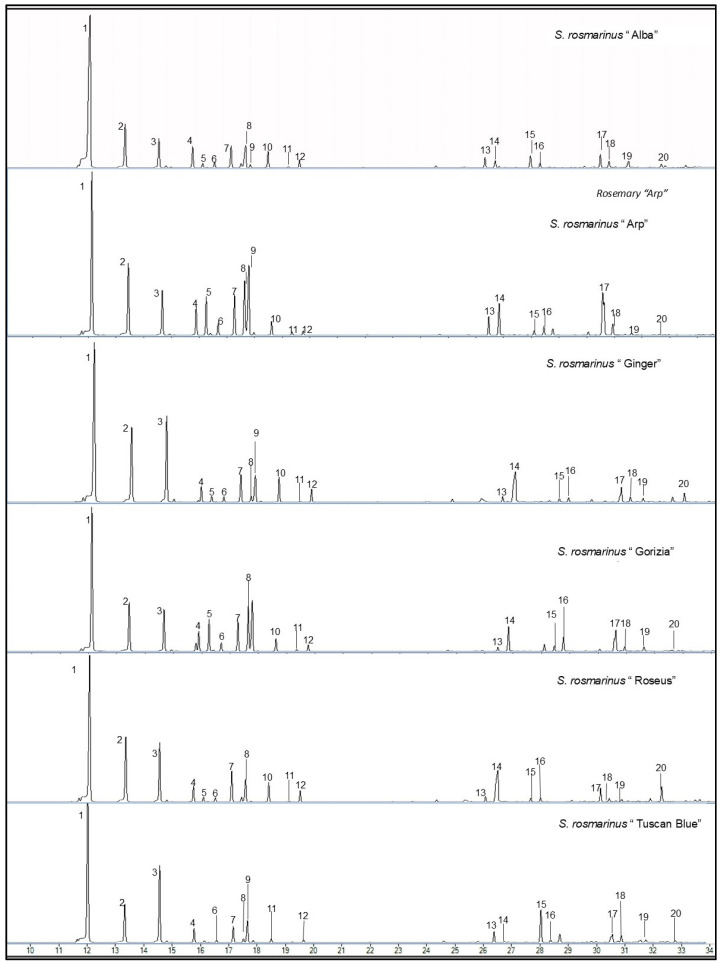
GC-MS chromatograms representative of the volatile terpenes identified and quantified for the six rosemary (*Salvia rosmarinus* Spenn.) cultivars, the peak number corresponds to the following sequence: **1**—α-Pinene; **2**—Camphene; **3**—β–Pinene; **4**—Myrcene; **5**—α-Phellandrene; **6**—α-Terpinene; **7**—D-Limonene; **8**—Cineolo; **9**—Ocimene; **10**—γ-Terpinene; **11**—p-Cymene; **12**—δ-Terpinene; **13**—Linalool; **14**—Camphor; **15**—Terpinen-4-ol; **16**—Caryophillene; **17**—α-Terpineol; **18**—(-)Borneol; **19**—Verbenone; **20**—β-Bisobolene.

The foliar terpene content of the rosemary cultivars was determined using gas chromatography-mass spectrometry (GC-MS) analysis (Figure 2), leading to the identification of 20 terpenes grouped into five major categories based on their chemical structure: sesquiterpenes (caryophyllene, β-bisabolene), monoterpenes (α-pinene, β-pinene), terpinene derivatives (α-terpinene, γ-terpinene, δ-terpinene), aliphatic terpenes (myrcene, ocimene), and terpenoids (cineole, terpinen-4-ol, linalool, camphor, verbenone, α-terpineol, (-)-borneol). Additionally, camphene, α-phellandrene, D-limonene, and p-cymene were analyzed separately (Table 2 and Table S2).

Statistical analyses revealed significant differences (*p* < 0.05) in the total terpene content among most of the rosemary cultivars (see Table 2). Specifically, ‘Alba’ exhibited the highest total terpene content (19.03 ± 0.88 µg/g DW), followed by ‘Ginger’ (17.43 ± 0.74 µg/g DW) and ‘Roseus’ (13.78 ± 0.74 µg/g DW). The lowest total terpene content was observed in ‘Arp’ and ‘Gorizia’, with values of 5.75 ± 0.43 µg/g DW and 6.33 ± 0.59 µg/g DW, respectively. However, no significant difference was found between the ‘Gorizia’ and ‘Arp’ cultivars.

The main terpenes across all cultivars were α/β-pinene and terpenoids, as shown in Table 2. The highest concentration of the α/β-pinene was found in the ‘Alba’ cultivar (9.13 ± 0.34 µg/g DW), followed by ‘Ginger’ (6.85 ± 0.11 µg/g DW), and ‘Roseus’ (5.32 ± 0.19 µg/g DW). Concerning the terpenoids, ‘Ginger’ exhibited the highest concentration (5.21 ± 0.35 µg/g DW), followed by ‘Alba’ (4.53 ± 0.18 µg/g DW) and ‘Roseus’ (4.12 ± 0.19 µg/g DW).

Significant quantitative differences were found for the individual terpenes. The α-phellandrene content in ‘Gorizia’ was significantly higher than the other cultivars. The ‘Ginger’ cultivar showed the highest camphene and terpenoids content. The ‘Alba’ cultivar was characterized by the highest α/β-pinene and aliphatic terpenes content. Furthermore, the ‘Roseus’ cultivar showed significant differences in D-limonene content.

**Figure 3:**
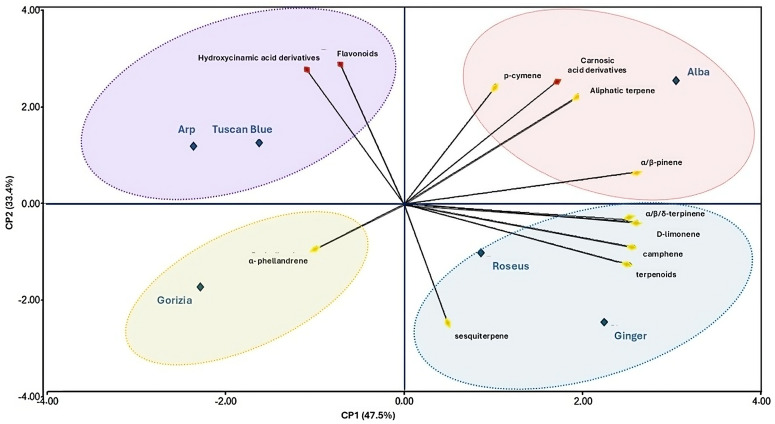
Principal component analysis-biplot of the rosemary (*Salvia rosmarinus* Spenn.) cultivars in relation to the polyphenol and terpene compounds. Blue diamonds represent ‘Tuscan Blue’, ‘Gorizia’, ‘Alba’, ‘Ginger’, ‘Roseus’, and ‘Arp’. Red diamonds represent the polyphenol classes (hydroxycinnamic acid derivatives, flavonoids, and phenolic diterpenes). Yellow diamonds represent terpenes (camphene, α-phellandrene, D-limonene, p-cymene, and the five classes of sesquiterpenes, terpenoids, α/β-pinene, α/ γ /δ-terpinene, and aliphatic terpenes).

A principal component analysis (PCA) was carried out to determine the most representative terpenes and phenolics in the rosemary cultivars, thereby identifying the correlated variables. The results showed two principal components that explain approximately 81% of the total variance, the first one (Axis X, CP1) representing 47.5%, and the second one (Axis Y, CP2) representing 33.4% (Figure 3).

The CP1 axis is mainly correlated with terpenes and phenolic compounds as follows: α/β-pinene (0.95), α/γ/δ-terpinene (0.94), camphene (0.93), terpenoids (0.91) and D-limonene (0.90). Regarding phenolic compounds and diterpenes, the class of carnosic acid derivatives exhibited a contribution of (0.62). CP2 is mainly explained by the following compounds: hydroxycinnamic acid derivatives (0.84), flavonoids (0.88), carnosic acid derivatives (0.76), and p-cymene (0.76). The PCA analysis identified four main clusters that divide the rosemary cultivars based on their terpene and phenol composition, revealing similarities between some of them. The first cluster, comprising the ‘Roseus’ and ‘Ginger’ cultivars, revealed a similarity in their terpene’s abundance, mainly characterized by α/γ/δ-terpinene, D-limonene, terpenoids, sesquiterpenes, and camphene. The second cluster comprises only the ‘Alba’ cultivar, which is characterized by a high content of terpenes, phenols, and diterpenes. The third cluster consists of ‘Tuscan Blue’ and ‘Arp’, which are similar in terms of their content of flavonoids and hydroxycinnamic acid derivatives. The last cluster is composed of the ‘Gorizia’ cultivar, characterized by high content of α-phellandrene and notably a low concentration of other phenols and diterpenes. This unique composition, different from the rest of the cultivars, may limit the applicability of ‘Gorizia’ in various fields.

### 2.3. Antioxidant Activity of Rosemary Extracts

The antioxidant activity of the phenolic extracts obtained from the rosemary cultivars was evaluated using FRAP and DPPH assays (Table 3). The results from the FRAP assay indicated that ‘Alba’ and ‘Tuscan Blue’ displayed the highest antioxidant activity, followed by ‘Ginger’ and ‘Roseus, which showed similar values.

The results of the FRAP assay for the terpene extracts indicated that ‘Ginger’ exhibited significantly higher antioxidant activity compared to all other cultivars. The DPPH assay of these extracts revealed that ‘Tuscan Blue’ had the greatest antioxidant activity of all the cultivars, followed by ‘Arp’. Additionally, ‘Gorizia’ and ‘Alba’ showed similar values. The ‘Roseus’ cultivar exhibited the lowest antioxidant activity.

### 2.4. Pearson’s Correlations Between Total Terpenes, Phenolics, and Antioxidant Activity

**Figure 4:**
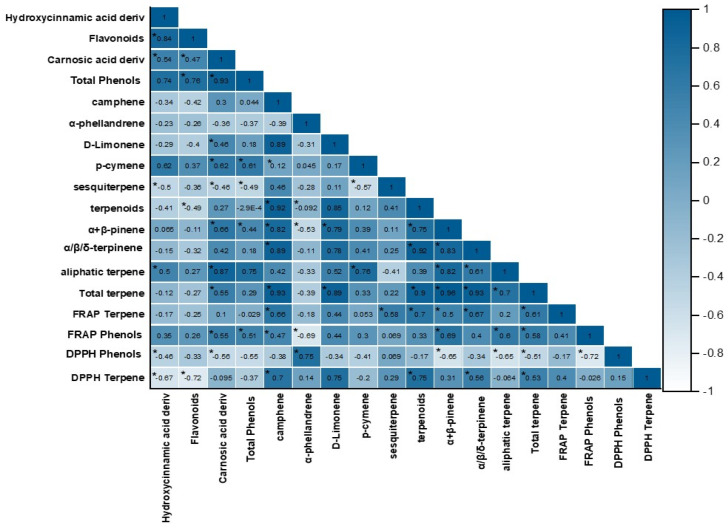
Pearson’s correlation coefficients (r) of hydroxycinnamic acid derivatives, flavonoids, phenolic diterpenes, and terpenes quantified in rosemary (*Salvia rosmarinus* Spenn.) cultivars versus FRAP and DPPH antioxidant activity. The asterisks indicate statistical significance (*p* < 0.05).

A potential correlation between the terpenes, polyphenols, and diterpenes identified and quantified in the rosemary cultivars and their antioxidant activity was analyzed using Pearson’s correlation (Figure 4). According to these results, significant correlations (*p* < 0.05) were found between total phenolic content and antioxidant activity, as assessed using FRAP (FRAP phenols: r = 0.51) and DPPH (DPPH phenols: r = −0.55). Regarding the most abundant phenolics, significant correlations were observed for carnosic acid derivatives content and the antioxidant activity of the phenolic extracts, assessed using FRAP (FRAP phenols: r = 0.55) and DPPH (DPPH phenols: r = −0.56) assays.

Significant correlations were found between total terpenes content and the antioxidant activity of the terpene extracts, analyzed using FRAP (FRAP terpenes: r = 0.61) and DPPH (DPPH terpenes: r = 0.53) assays. Some of the most abundant terpenes extracted showed significant correlations with antioxidant activity, evaluated using FRAP assay, as follows: α+β-pinene (r = 0.51), terpenoids (r = 0.70), camphene (r = 0.66), and limonene (r = 0.44).

## 3. Discussion

The quality of rosemary (*Salvia rosmarinus* Spenn.) is strictly related to the content of high-value phytochemicals, such as phenols and terpenes, influencing its potential applications [19,26,27,28,29]. A comprehensive characterization of these compounds, obtained through chemical analysis and specific assays for the evaluation of their antioxidant capacity, can lead to more reliable results and consistent conclusions.

Among the multiple bioactivities attributed to rosemary, the antioxidant one is of remarkable interest, as antioxidant species protect the cell from oxidative damage, thus preventing its degeneration [30]. The ability of rosemary chemical compounds to chelate metal ions (such as Fe²⁺), that can induce oxidative stress, and more broadly, to enhance the production of antioxidant enzymes, plays a key role in the regulation of various metabolic pathways [31,32,33]. 

Overall, the antioxidant activity of rosemary might be attributed to the combined action of its flavonoids and phenolics diterpenes compounds, as well as to the ratio between carnosic acids and carnosol [24]. The carnosic acid derivatives were the most abundant phenolic compounds across all the rosemary cultivars considered in this work, as previously reported for other *S. rosmarinus* cultivars [5,34,35]. Notably, carnosic acid, which is a phenolic diterpene, has been identified as the main component in the young leaves of rosemary [25,36,37,38], explaining the dominance of this compound and its derivatives in all the considered cultivars.

The ‘Alba’ cultivar was found to be the richest in carnosic acid derivatives, showing significantly higher values than all the other cultivars. Carnosic acid is well-known for its strong antioxidant activity, attributed to its chemical structure, specifically the catechol unit with hydroxyl groups in the orthoposition (C_11_–C_12_), which is directly related to its effectiveness as an antioxidant [5,39]. In this context, significant and moderate Pearson’s correlations were obtained for the antioxidant activity measured using FRAP and DPPH assays of this diterpene.

On the other hand, among the hydroxycinnamic acids, rosmarinic acid was identified as the leading compound, which is consistent with the composition of rosemary extracts reported in previous investigations [37]. In our study, the greatest amount of rosmarinic acid was found in ‘Arp’ and ‘Tuscany Blue’, which were also characterized by the highest content of flavonoids. Plants synthesize hydroxycinnamic acids as a protection mechanism against biotic and abiotic stresses (microbial infection, high solar radiation); however, these compounds have also been found to play an important protective role in humans due to their antioxidant, antimicrobial, and anti-inflammatory properties [4,5]. Thus, based on their hydroxycinnamic acids content and in vitro antioxidant activities, efficient performances in terms of anti-inflammatory activity could be expected from the ‘Tuscan Blue’ and ‘Arp’ cultivars [40].

The class of flavonoids has been widely investigated for its anticarcinogenic properties [41]. Nevertheless, no significant correlations were obtained between the antioxidant activity and flavonoids. Although higher antioxidant activity of the diterpenes (carnosol, carnosic acid and its derivatives) compared to the flavonoids has been reported [42,43,44], it is not possible to conclude or attribute superior antioxidant activity to a specific class of compounds. In addition, the ratio between carnosic acids and carnosol can affect the antioxidant activity since, while carnosic acid is more effective than carnosol, the latter is likely to react faster with DPPH radicals than carnosic acids [24]. The antioxidant activity of phenolic extracts should rather be considered as resulting from the synergy of all the compounds present within a cultivar, as well as the ratio between some of these compounds. As the phenolic composition of rosemary extracts may vary based on the sampling season, plant phenology, agronomical, and processing conditions, the quantification of the single compounds in rosemary extracts should be accurately conducted to obtain a holistic picture of the technological applications of different rosemary cultivars [15,16]. In our study, given the significant correlations observed between the antioxidant activity measured using FRAP and DPPH assays, and the total phenol content, ‘Alba’, which exhibits the highest content of these compounds, could be suggested to be the ideal candidate for antioxidant applications.

Considering that the highest number of plant secondary metabolites belongs to the terpenes, this class is widely investigated for its antioxidant, anti-inflammatory [45], antibacterial, and antiviral properties [34,46], along with applications in the pharmaceutical industry [47]. The analysis of the six rosemary cultivars revealed that α- and β-pinene were the most abundant terpenes in all of them, followed by the terpenoids. In agreement with these results, α- and β-pinene have been previously reported as the major components of rosemary extracts [17,48]. Moreover, both the pinenes showed significant and moderate Pearson correlations with the antioxidant activity measured using FRAP and DPPH assays, highlighting their valuable contribution to the antioxidant properties of the rosemary cultivars. The antioxidant potential of these terpenes and other terpenoids is related to their hydroxylated structure [49,50]. Among the six cultivars, ‘Alba’ showed the highest content of α/β-pinene, *p*-cymene, and aliphatic terpenes. Likewise, in ‘Ginger’ and ‘Roseus’, the most predominant compounds were α/β-pinene, terpenoids, and camphene, which are strictly correlated with high antioxidant activity as measured by the FRAP (*r* = 0.66) and DPPH (*r* = 0.70) assays. Terpenoids showed a high correlation with antioxidant activity measures (FRAP terpenoids: *r* = 0.70). Furthermore, sesquiterpenes showed a significant moderate relationship with the FRAP assay (*r* = 0.58). Overall, as expected, total terpenes showed a significant correlation with antioxidant activity (FRAP, *r* = 0.61; DPPH, *r* = 0.53), suggesting that cultivars with high concentrations of these compounds may possess strong antioxidant potential.

The PCA analysis of the rosemary cultivars lead to the discrimination of four clusters, revealing that ‘Arp’ and ‘Tuscan Blue’ were very similar, as well as ‘Roseus’ and ‘Ginger’, whereas ‘Alba’ and ‘Gorizia’ were clearly distinct, originating two separate clusters. Particularly, the ‘Alba’ cluster appeared to be completely distinct from all the others, indeed, turning out to be the richest in terpenes and phenols among all the cultivars. The characterization of specific cultivars, based on the content of their secondary metabolites, is of remarkable interest in view of the targeted selection of plant material for technological applications. In this study, the different rosemary cultivars were grown under the same environmental conditions thus providing valuable information on rosemary chemical profiles whose differences may be attributed only to the cultivars rather than other ecological and agronomical factors.

In conclusion, on the basis of these results, ‘Alba’ stands out as the cultivar with the optimal composition in terms of antioxidant constituents, appearing as a promising candidate for various applications in the pharmaceutical, nutraceutical, and cosmetic industries. Genetic differences in rosemary cultivars not only influence flower color and leaf shape, but also play a key role in the biosynthesis and variability of both phenolic and terpene content, determining possible different technological applications. Given the importance of the agronomical factors on plant secondary metabolism, future research may investigate whether moderate stress applied during the cultivation in field conditions may increase or alter the phenolic and terpene composition of leaf extracts of these six Mediterranean rosemary cultivars.

## 4. Materials and Methods

### 4.1. Sampling of Foliar Tissue of Rosemary Cultivars

Leaves collected from branches of four-year-old plants (propagated from cuttings) of six *Salvia rosmarinus* Spenn. syn. *Rosmarinus officinalis* L. cultivars, named ‘Alba’, ‘Arp’ ‘Ginger’, ‘Gorizia’, ‘Tuscan Blue’, and ‘Roseus’, were utilized in this study. Leaf samples were collected in January 2021, during the morning, from potted plants belonging to the cultivation area of the GEA Park—Green Economy and Agriculture Park, located in Pistoia, Italy (43°55′9.077″ N 10°54′25.913″ E). The plants were cultivated as potted plants grown outdoors filled with commercial soil and maintained under field conditions in the common garden of GEA park, with provisions for on-demand irrigation and natural lighting. All cultivars were grown under the same conditions following the standard protocols of the nursery, and ten plants per cultivar were used as source of leaves. To extract the different metabolites (terpenes and phenolics), branches measuring 30–40 cm from different positions of the shrubs were manually harvested and frozen and stored in liquid nitrogen until reaching the laboratory.

### 4.2. Analysis of Phenolic and Phenolic Diterpenes Compounds

Once in the laboratory, the frozen leaves were ground (70 mg) and extracted using 3 × 1.8 mL ethanol 75% (pH 2.5 adjusted with formic acid) through Ultrasound-Assisted Extraction (UAE). The UAE was performed in an ultrasonic bath (BioClass^®^ CP104, 39 kHz, and 100 W of input power), at a low temperature (approx. 4–6 °C) for 30 min. After the extraction, the samples were centrifuged (5 min, 9000 rpm, 5 °C—ALC^®^ 4239R, Milan, Italy) and the supernatants were defatted by n-hexane extraction (3 × 1.8 mL). The defatting with n-hexane was performed to remove lipophilic compounds (mainly chlorophylls and carotenoids). The ethanol extracts were then evaporated to dryness using a speed-vac concentrator (Eppendorf^®^ Concentrator Plus) for 2 h. Subsequently, the extracts were resuspended with 200 μL of methanol–water solution (50% *v*/*v*, pH 2.5 adjusted with formic acid) and analyzed using HPLC-DAD.

Aliquots (10 µL) of the samples were injected into a Perkin^®^ Elmer Flexar liquid chromatograph equipped with a quaternary 200Q/410 pump and an LC 200 diode array detector (DAD) (all from Perkin Elmer^®^, Bradford, CT, USA). The mobile phase consisted of a gradient of (A) acidified water (at pH 2.5 adjusted with formic acid) and (B) acetonitrile (at pH 2.5 adjusted with formic acid), while the stationary phase consisted of an Agilent^®^ Zorbax^®^ SB-18 column (250 × 4.6 mm, 5 µm), kept at 30 °C. The following gradient was applied: 0–1 min (3% B), 1–56 min (3–40% B), 56–61 min (40% B), 61–69 min (40–97% B), 69–79 min (97–3% B), and the flow elution was 0.6 mL min^−1^.

The chromatograms were obtained at 280, 330, and 350 nm. The identification of the most prevalent phenolic compounds was conducted through detailed analysis of the UV spectra and comparison with the following standards: rosmarinic acid, caffeic acid, hesperidin, quercentin 3-O-glucoside, luteolin 7-O-glucoside, apigenin 7-O-glucoside, carnosol, and carnosic acid (Figures S1–S8). For the accurate analysis of quercetin, luteolin, and apigenin derivatives, retention times and elution order reported in the existing literature on rosemary extracts were considered [51,52]. Quantification was subsequently carried out using calibration curves of each standard based on five-point measurements. The quantitative results for the phenolic compounds were expressed as the total of hydroxycinnamic acid derivatives, carnosic acid derivatives, and flavonoids. Each of them corresponds to the sum of the content of all the individual compounds within each specific class while the total phenolic content is the result of the sum of all the classes. The results were expressed on a dry weight (DW) basis, utilizing the fresh weight to dry weight (FW/DW) ratio.

### 4.3. GC-MS Analysis of Terpene Content in Rosemary Cultivars

Frozen rosemary leaves were subjected to liquid extraction to determine the terpene content using gas chromatography-mass spectrometry (GC-MS) analysis. One mL of heptane tridecane (20 ppm) was added to 0.5 g of leaf material into a 2 mL glass vial. The sample underwent 3 cycles of sonication (15 min each) and was kept under agitation for 24 h at a constant temperature of 35 °C. Following centrifugation (4000 rpm; 10 min), 100 μL of the extract was collected for GC-MS analysis. The analysis was conducted using an Agilent 7820A Gas Chromatograph (GC) equipped with a single quadrupole Mass Selective Detector (MSD) model 5977E (both from Agilent Technology, Palo Alto, CA, USA). A volume of 1 μL of extract in heptane tridecane was injected in a split/splitless injector operating in splitless mode. A Gerstel MPS2 XL autosampler (Gerstel, Mülheim an der Ruhr, Germany) equipped with a liquid option was used. Helium served as the carrier gas (pressure: 33 psi; flow: 1.2 mL/min). The chromatographic conditions were as follows: the injector temperature was set at 260 °C in splitless mode, utilizing an Agilent DB-Wax UI column (60 m × 0.25 mm × 0.5 μm). The oven temperature program: initial temperature 40 °C for 1 min; 5 °C per min until 200 °C; 10 °C per min until 240 °C holding this time for 6 min. The mass detector operated with an electron ionization of 70 eV and was set in scan mode in the *m*/*z* range of 40–350, at a rate of three scans per second. Data were analyzed using the Agilent Mass Hunter software Workstation (Qualitative Analysis-Version B.06.00 and Quantitative Analysis Version B.07.01/Build 7.1.524.0) [53]. Peak identification was confirmed by authentic standards analyzed under the same conditions and by matching their mass spectra and retention times with those reported in the National Institute of Standards and Technology (NIST 11, Gaithersburg, MD, USA) spectral database library. Data are expressed as means of three replicates (SD < 5%). The results are expressed on a dry weight (DW) basis using the fresh weight to dry weight (FW/DW) ratio and calculated for each cultivar.

### 4.4. Antioxidant Activity Assays of Rosemary Extracts

#### 4.4.1. DPPH Radical-Scavenging Activity Assay

The DPPH (2,2-diphenyl-1-picrylhydrazil) assay was conducted following the protocol optimized reported elsewhere [54]. Concisely, 250 μL of diluted samples (10 μL of the extracts diluted in 5000 μL of MeOH) were added at 250 μL of a DPPH solution (0.1 mM in methanol; sourced from Sigma Aldrich, Merck, KGaA, Darmstadt, Germany). Rosmarinic acid (Sigma Aldrich, Merck, KGaA, Darmstadt, Germany) was used as the positive control. The mixture was then incubated in the dark at room temperature for 45 min. Absorbance measurements were taken at 518 nm using a Perkin Elmer^®^ UV/Vis spectrophotometer (Lambda 25, Perkin Elmer, Bradford, CT, USA). Following this, the absorbances for the blank (250 μL methanol and 250 μL of diluted sample) and the negative controls (250 μL of MeOH (added with the same proportion of solvent of extraction as in the diluted samples) and 250 μL DPPH solution) were recorded. Three replicates were performed on the two extracts, phenolic and terpene extracts (prepared as reported in Section 4.2 and Section 4.3), for each cultivar. The EC_50_ (effective concentration at 50%) values were calculated by linear regression of plots where the abscissa represented the concentration of tested plant extracts and the ordinate the average percent of antioxidant activity from three separate tests. The percentage of antioxidant activity was calculated using the formula:

AA% = 100 − {[(ABS*_sample_* − ABS*_blank_*) × 100]/ABS*_negative control_*}

#### 4.4.2. Ferric Reducing Antioxidant Power (FRAP) Assay

The FRAP assay is based on the reduction of ferric 2,4,6-tris(2-pyridyl)-1,3,5-triazine [Fe(III)-TPTZ] to the ferrous complex [Fe(II)-TPTZ] at an acidic pH, developing an intense blue color. This change is quantified by measuring the absorbance at 594 nm using a spectrophotometer. The assay was conducted using a FRAP assay kit (Sigma Aldrich, Milan, Italy). Briefly, 10 µL of sample (rosemary phenolic and terpene extracts of each cultivar) was added with 152 µL of buffer, 19 µL of FeCl_3_, and 19 µL of FRAP probe. Blanks were prepared with the same solvents of the extracts without the FRAP probe. After incubating for 60 min at 37 °C, the absorbance at 594 nm was measured using a SpectraMax^®^ Microplate reader (Molecular Devices, San Jose, CA, USA). Calibration was performed with Fe(II) (FeSO_4_·7H_2_O) solutions ranging from 0 to 20 nmol and the assay was carried out in triplicate. The standard curve was linear between the concentrations of FeSO_4_ used_,_ and an ascorbic acid solution (Sigma Aldrich, Merck, KGaA, Darmstadt, Germany) was used as the positive control.

### 4.5. Statistical Analysis

To test data normality, the Kolmogorov–Smirnov test was performed. For those data following a normal distribution, a one-way ANOVA was carried out, combined with the Duncan and Tukey post hoc tests. The differences between the means were considered significant for *p*-values < 0.05. For data not following a normal distribution, the non-parametric Kruskal–Wallis test was applied. Additionally, a principal component analysis was performed to identify the most represented terpenes and phenols in each rosemary cultivar. Finally, a Pearson correlation test was conducted to assess the strength of the association between the phenols/terpenes content and antioxidant activity. All statistical tests were performed using SPSS Statistics, Version 29.0.2.0 [55].

## Figures and Tables

**Table 1:** Hydroxycinnamic acid derivatives, flavonoids, and phenolic diterpenes (mg/g of dry weight) in the six rosemary (*Salvia rosmarinus* Spenn.) cultivars. Values are reported as mean ± standard deviation of three replicates. Means with the same letter are not significantly different (Duncan test; *p* > 0.05). The ratio between carnosic acids and carnosol is also reported in the table.

*S. rosmarinus* Cultivars						
Class	‘T. Blue’	‘Gorizia’	‘Alba’	‘Ginger’	‘Roseus’	‘Arp’
Hydroxycinnamic acid derivatives	1.08 ± 0.28 ^b^	0.38 ± 0.045 ^c^	0.82 ± 0.22 ^b^	0.23 ± 0.09 ^c^	0.64 ± 0.078 ^b^	1.83 ± 0.10 ^a^
Flavonoids	6.36 ± 1.43 ^a^	2.92 ± 0.92 ^b^	5.32 ± 1.51 ^a^	1.72 ± 0.68 ^b^	1.89 ± 0.67 ^b^	4.98 ± 1.07 ^a^
Phenolic diterpenes	14.91 ± 1.47 ^b^	12.00 ± 2.57 ^c^	24.85 ± 2.72 ^a^	14.55 ± 1.49 ^b^	17.13 ± 3.32 ^b^	15.44 ± 5.35 ^b^
Total polyphenols	22.35 ± 3.18 ^b^	15.30 ± 3.54 ^b^	30.99 ± 4.45 ^a^	17.35 ± 2.35 ^b^	19.65 ± 4.60 ^b^	22.25 ± 6.51 ^ab^
Carnosic acid derivatives/Carnosol	2.35 ± 0.14	4.62 ± 0.21	4.38 ± 0.89	93.47 ± 3.01	9.82 ± 1.65	3.79 ± 0.38

**Table 2:** Terpene composition and content (µg/g DW) in the six rosemary (*Salvia rosmarinus* Spenn.) cultivars. Values are reported as mean ± standard deviation of three replicates. Means with the same letter are not significantly different by Duncan test (*p* > 0.05).

	*S. rosmarinus* Cultivars					
Compounds	‘T. Blue’	Gorizia’	‘Alba’	‘Ginger’	‘Roseus’	‘Arp’
Camphene	0.988 ± 0.115 ^bc^	0.640 ± 0.095 ^c^	2.359 ± 0.099 ^ab^	3.097 ± 0.029 ^a^	2.438 ± 0.173 ^ab^	0.794 ± 0.019 ^bc^
α-Phellandrene	-	0.224 ± 0.018 ^a^	0.092 ± 0.003 ^c^	0.089 ± 0.003 ^c^	0.053 ± 0.011 ^d^	0.152 ± 0.009 ^b^
D-Limonene	0.373 ± 0.028 ^c^	0.410 ± 0.060 ^c^	1.140 ± 0.053 ^a^	0.958 ± 0.086 ^b^	1.208 ± 0.050 ^a^	0.374 ± 0.066 ^c^
*p*-Cymene	0.070 ± 0.009 ^d^	0.024 ± 0.005 ^e^	0.323 ± 0.027 ^a^	0.109 ± 0.011 ^c^	0.025 ± 0.004 ^e^	0.237 ± 0.022 ^b^
sesquiterpenes	0.030 ± 0.004 ^b^	0.022 ± 0.002 ^c^	0.006 ± 0.001 ^e^	0.055 ± 0.004 ^a^	0.029 ± 0.003 ^b^	0.013 ± 0.002 ^d^
α/β-Pinene	4.579 ± 0.432 ^d^	1.668 ± 0.207 ^e^	9.127 ± 0.338 ^a^	6.854 ± 0.109 ^b^	5.318 ± 0.193 ^c^	1.458 ± 0.080 ^e^
α/γ/δ-Terpinene	0.099 ± 0.005 ^e^	0.181 ± 0.034 ^d^	0.770 ± 0.101 ^b^	0.819 ± 0.007 ^a^	0.386 ± 0.072 ^c^	0.190 ± 0.022 ^d^
Aliphatic terpenes	0.273 ± 0.018 ^b^	0.083 ± 0.004 ^d^	0.683 ± 0.062 ^a^	0.244 ± 0.023 ^bc^	0.204 ± 0.006 ^c^	0.197 ± 0.018 ^c^
Terpenoids	2.286 ± 0.372 ^e^	3.079 ± 0.170 ^d^	4.527 ± 0.176 ^b^	5.210 ± 0.346 ^a^	4.119 ± 0.188 ^c^	2.339 ± 0.099 ^e^
Total terpenes	8.708 ± 0.983 ^d^	6.331 ± 0.595 ^d^	19.028 ± 0.877 ^a^	17.434 ± 0.740 ^b^	13.779 ± 0.744 ^c^	5.753 ± 0.429 ^e^

**Table 3:** Antioxidant activity of rosemary (*Salvia rosmarinus* Spenn.) extracts assessed using FRAP and DPPH assays. Means with the same letter are not significantly different (Duncan test; *p* > 0.05).

Cultivar	Phenolic Extracts		Terpene Extracts	
	FRAP (mM Fe(II)/mg)	DPPH (EC_50_, µg/mL)	FRAP (mM Fe(II)/mg)	DPPH (EC_50_, µg/mL)
‘Tuscan Blue’	9.374 ± 1.554 ^b^	0.660 ± 0.010 ^c^	11.699 ± 1.561 ^c^	0.210 ± 0.002 ^e^
‘Gorizia’	6.469 ± 0.691 ^d^	0.940 ± 0.0130 ^a^	11.260 ± 1.513 ^c^	0.529 ± 0.010 ^c^
‘Alba’	9.766 ± 1.028 ^b^	0.630 ± 0.060 ^c^	13.185 ± 1.762 ^c^	0.528 ± 0.066 ^c^
‘Ginger’	8.659 ± 0.823 ^bc^	0.750 ± 0.0100 ^bc^	16.677 ± 2.580 ^b^	0.690 ± 0.067 ^b^
‘Roseus’	8.485 ± 0.739 ^bc^	0.750 ± 0.020 ^bc^	12.325 ± 1.645 ^c^	0.784 ± 0.025 ^a^
‘Arp’	7.166 ± 0.761 ^c^	0.800 ± 0.030 ^bc^	11.072 ± 1.513 ^c^	0.393 ± 0.034 ^d^
Positive control	61.258 ± 5.923 ^a^	0.100 ± 0.012 ^d^	61.258 ± 5.923 ^a^	0.100 ± 0.012 ^f^

## Data Availability

Data are contained within the article and Supplementary Materials.

## Supplementary Materials

The following supporting information can be downloaded at: https://www.mdpi.com/article/10.3390/plants13233395/s1. Table S1. Compounds extracted and quantified in rosemary cultivars, classified by chemical group: Hydroxycinnamic acid derivatives, Flavonoids and Phenolic diterpenes (means ± standard deviation, *n* = 3). Table S2. Terpenes extracted and quantified in rosemary cultivars, classified by chemical group: Sesquiterpene, Terpenoids, α/β-pinene, Aliphatic terpene and α/γ/δ-terpinene. The compounds camphene, α-phellandrene, D-limonene, *p*-cymene have been individually quantified (means ± standard deviation, *n* = 3). Figure S1. Chromatogram of Caffeic acid standard RT = 29.95 min, and UV spectrum. Standard used for the quantification peak as follows: 1.-Caffeic acid; 2.-Caffeic acid-deriv. Figure S2. Chromatogram of Quercentin standard RT = 37.42 min, and UV spectrum. Standard used for the quantification peak as follows: 3.-Quecerin deriv-1; 4.-Quecerin deriv-2; 6.-Quecerin deriv-3. Figure S3. Chromatogram of Luteolin standard RT = 38.81 min, and UV spectrum. Standard used for the quantification peak as follows: 5.-Luteolin-deriv-1; 10.-Luteolin-deriv-2; 12.-Luteolin-deriv-3; 13.-Luteolin-deriv-4; 15.-Luteolin-deriv-5; 16.-Luteolin-deriv-6; 17.-Luteolin-deriv-7. Figure S4. Chromatogram of Hesperidin standard RT = 40.98 min, and UV spectrum. Figure S5. Chromatogram of Apegenin standard RT = 41.89 min, and UV spectrum. Standard used for the quantification peak as follows: 8.-Apigenin-deriv-1; 11.-Apigenin-deriv-2; 14.-Apigenin-deriv-3. Figure S6. Chromatogram of Rosmarinic acid standard RT = 43.37 min, and UV spectrum. Standard used for the quantification peak as follows: 9.-Rosmarinic acid; 18.-Rosmarinic acid deriv-1. Figure S7. Chromatogram of Carnosol standard RT = 71.11 min, and UV spectrum. Figure S8. Chromatogram of Carnosic Acid standard RT = 74.46 min, and UV spectrum. Standard used for the quantification peak as follows: 20.-Carnosic acid; 21.-Carnosic acid deriv-1; 22.-Carnosic acid deriv-2.
