# Supplementary material for: Unlocking the Hidden Potential of Rosemary (Salvia rosmarinus Spenn.): New Insights into Phenolics, Terpenes, and Antioxidants of Mediterranean Cultivars
Source: Plants (Basel). 2024 Dec 3;13(23):3395. doi: 10.3390/plants13233395 (PMC11644807; doi:10.3390/plants13233395)
Supplement: Supplementary file 1 [file plants-13-03395-s001.zip › plants-3275231-supplementary.pdf]

Table S1. Compounds extracted and quantified in rosemary cultivars, classified by chemical group: Hydroxycinnamic acid derivatives, Flavonoids and Phenolic diterpenes (means ± standard deviation, n=3).

			S. rosmarinus cultivars					
**Peak**	**Class**	**R_t (min)**	**'T. Blue'**	**'Gorizia'**	**'Alba'**	**'Ginger'**	**'Roseus'**	**'Arp'**
			Hydroxycinnamic acid derivatives					
**1**	Caffeic acid	29.95	0.054± 0.003^{a}	0.028 ±0.012^{bcd}	0.029±0.009^{cd}	0.030±0.009^{c}	0.027±0.007^{bcd}	0.035±0.006^{b}
**2**	Caffeic acid der	36.78	0.006±0.003^{c}	0.003±0.002^{c}	0.019±0.008^{a}	0.004±0.004^{c}	0.007±0.007^{c}	0.014±0.011^{ab}
**9**	Rosmarinic acid	43.37	0.987±0.259^{b}	0.340±0.030^{d}	0.745±0.198^{bc}	0.194±0.081^{d}	0.602±0.063^{cd}	1.761±0.070^{a}
**18**	Rosmarinic derv-1	69.90	0.033±0.015^{ab}	0.005±0.001^{bd}	0.023±0.009^{ab}	0.001±0.000^{bd}	0.003±0.001^{bd}	0.017±0.013^{a}
	**Total**		**1.080±0.280^{b}**	**0.377±0.045^{c}**	**0.816±0.224^{b}**	**0.229±0.094^{c}**	**0.640±0.078^{b}**	**1.827±0.101^{a}**
			Flavonoids					
**3**	Quercetin deriv-1	37.42	0.402±0.049^{a}	0.034±0.003^{d}	0.126±0.038^{bc}	0.016±0.011^{d}	0.013±0.004^{d}	0.155±0.027^{b}
**4**	Quercetin deriv-2	38.19	0.085±0.034^{c}	0.058±0.016^{c}	0.448±0.088^{ab}	0.025±0.012^{c}	0.016±0.009^{c}	0.388±0.031^{b}
**5**	Luteolin deriv-1	38.81	0.081±0.035^{ns}	0.073±0.061^{ns}	0.131±0.030^{ns}	0.068±0.054^{ns}	0.038±0.026^{ns}	0.077±0.019^{ns}
**6**	Quercentin deriv-3	40.13	0.150±0.063^{a}	0.099±0.018^{ad}	0.110±0.041^{acd}	0.083±0.018^{ad}	0.057±0.003^{bd}	0.139±0.041^{ab}
**7**	Hesperidin	40.98	1.698±0.420^{ns}	0.755±0.217^{ns}	1.046±0.545^{ns}	0.779±0.190^{ns}	0.421±0.330^{ns}	1.206±0.751^{ns}
**8**	Apigenin deriv-1	41.89	0.110±0.013^{d}	0.156±0.027^{bc}	0.513±0.049^{a}	0.086±0.018^{cd}	0.119±0.040^{d}	0.215±0.016^{b}
**10**	Luteolin deriv-2	44.66	0.361±0.132^{a}	0.195±0.002^{ab}	0.100±0.037^{bc}	0.044±0.026^{c}	0.073±0.069^{c}	0.086±0.001^{c}
**11**	Apigenin deriv-2	46.53	0.047±0.024^{ab}	0.077±0.012^{a}	0.028±0.011^{c}	0.006±0.002^{c}	0.004±0.002^{c}	0.033±0.008^{c}
**12**	Luteolin deriv-3	47.01	0.921±0.168^{a}	0.455±0.163^{b}	0.431±0.129^{bc}	0.104±0.108^{c}	0.310±0.035^{c}	0.739±0.049^{ab}
**13**	Luteolin deriv-4	47.16	0.915±0.158^{a}	0.290±0.106^{bc}	0.350±0.100^{bc}	0.139±0.058^{c}	0.260±0.026^{c}	0.528±0.019^{b}
**14**	Apigenin deriv 3	47.68	0.205±0.076^{bc}	0.320±0.105^{b}	0.697±0.180^{a}	0.103±0.080^{bd}	0.140±0.011^{bd}	0.199±0.029^{bd}
**15**	Luteolin deriv-5	48.54	0.921±0.168^{a}	0.239±0.110^{d}	0.631±0.129^{bc}	0.157±0.055^{d}	0.310±0.035^{d}	0.739±0.049^{ab}
**16**	Luteolin deriv-6	49.63	0.316±0.041^{a}	0.037±0.011^{cd}	0.075±0.007^{c}	0.014±0.007^{d}	0.034±0.004^{cd}	0.202±0.011^{b}
**17**	Luteolin deriv-7	57.49	0.145±0.046^{c}	0.127±0.070^{c}	0.629±0.127^{a}	0.097±0.038^{c}	0.094±0.007^{c}	0.271±0.014^{b}
	**Total**		**6.36 ±1.43^{a}**	**2.92±0.92^{b}**	**5.32±1.51^{a}**	**1.72±0.68^{b}**	**1.89±0.67^{b}**	**4.98±1.07^{a}**
			Phenolic diterpenes					
**19**	Carnosol	71.11	4.461± 0.543^{ab}	2.136 ± 0.894^{d}	4.732± 1.179^{a}	0.154 ± 0.018^{e}	1.583 ± 0.412^{cd}	3.222 ± 0.725^{abc}
**20**	Carnosic acid	74.46	6.627 ± 0.412^{bd}	5.595± 0.492^{de}	11.542 ± 1.214^{a}	8.872 ± 1.001^{b}	7.871 ± 1.150^{bd}	6.622 ± 1.159^{be}
**21**	Carnosic acid der 1	76.54	1.029 ± 0.239^{bc}	1.231± 0.444^{abc}	1.989 ± 0.266^{ab}	1.839 ± 0.282^{b}	2.397 ± 0.241^{a}	1.478 ± 0.847^{abc}
**22**	Carnosic acid der 2	77.28	2.792 ±0.274^{d}	3.038± 0.744^{d}	6.580 ± 1.344^{a}	3.684 ± 0.187^{c}	5.274 ± 1.514^{ab}	4.119 ± 2.617^{bc}
	**Total**		**14.91 ± 1.47^{b}**	**12.00 ± 3.57^{c}**	**24.84 ± 4.00^{a}**	**14.55 ± 1.49^{b}**	**17.13 ± 3.32^{b}**	**15.44 ± 5.35^{b}**

Table S2. Terpenes extracted and quantified in rosemary cultivars, classified by chemical group: Sesquiterpene, Terpenoids, α/β-pinene, Aliphatic terpene and α/γ/δ-terpinene. The compounds camphene, α-phellandrene, D-limonene, *p*-cymene have been individually quantified (means ± standard deviation, n=3).

			S. rosmarinus cultivars					
**Peak**	**Class**	**R_t (min)**	**'T. Blue'**	**'Gorizia'**	**'Alba'**	**'Ginger'**	**'Roseus'**	**'Arp'**
**2**	Camphene	13.45	0.998±0.115^{bc}	0.640±0.095^{c}	2.359±0.099^{ab}	3.097±0.029^{a}	2.438±0.173^{ab}	0.794±0.019^{bc}
**5**	α-phellandrene	16.25	-	0.224±0.018^{a}	0.092±0.003^{c}	0.089±0.003^{c}	0.053±0.011^{d}	0.152±0.009^{b}
**7**	D-limonene	17.28	0.373±0.028^{c}	0.410±0.060^{c}	1.140±0.053^{a}	0.958 ± 0.086^{b}	1.208 ± 0.050^{a}	0.374 ± 0.066^{c}
**11**	*p*-cymene	19.35	0.070 ± 0.009^{d}	0.024 ± 0.005^{e}	0.323 ± 0.027^{a}	0.109 ±0.011^{c}	0.025 ± 0.004^{e}	0.237 ± 0.022^{b}
				Sesquiterpenes				
**20**	β-bisobolene	32.40	0.003±0.000^{c}	0.003±0.001^{c}	0.003±0.0001^{c}	0.039±0.003^{a}	0.027±0.003^{b}	0.004±0.001^{c}
**16**	Caryophillene	28.77	0.027±0.004^{a}	0.019±0.001^{b}	0.003±0.001^{cd}	0.016±0.001^{b}	0.002±0.000^{c}	0.009±0.002^{e}
	**Total**		**0.030±0.004^{b}**	**0.022±0.002^{c}**	**0.006±0.001^{e}**	**0.055±0.004^{a}**	**0.029±0.003^{b}**	**0.013±0.003^{d}**
				Terpenoids				
**8**	Cineol	17.77	1.816±0.333^{de}	2.498±0.125^{b}	2.948±0.097^{be}	3.513±0.256^{a}	2.795±0.155^{c}	1.588±0.079^{e}
**13**	Linalool	26.46	0.048±0.007^{b}	0.019±0.001^{f}	0.072±0.004^{a}	0.027±0.002^{d}	0.022±0.004^{e}	0.041±0.008^{c}
**14**	Camphor	26.82	0.016±0.001^{e}	0.297±0.030^{dc}	0.290±0.025^{dc}	1.351±0.065^{a}	1.142±0.046^{b}	0.366±0.041^{c}
**15**	Terpinen-4-ol	28.69	0.012±0.002^{d}	0.012±0.001^{e}	0.045±0.001^{a}	0.017±0.001^{c}	0.019±0.001^{bc}	0.021±0.002^{b}
**17**	α-terpineol	30.62	0.050±0.005^{ce}	0.066±0.002^{d}	0.129±0.005^{a}	0.101±0.001^{b}	0.059±0.001^{d}	0.072±0.005^{c}
**18**	(-)borneol	30.94	0.254±0.021^{b}	0.085±0.010^{d}	0.396±0.018^{a}	0.180±0.019^{c}	0.046±0.004^{d}	0.210±0.012^{c}
**19**	Verbenone	31.71	0.090±0.003^{c}	0.102±0.001^{bc}	0.647±0.033^{a}	0.020±0.002^{d}	0.036±0.002^{d}	0.040±0.004^{d}
	**Total**		**2.286±0.372^{e}**	**3.079±0.170^{d}**	**4.527±0.183^{b}**	**5.209±0.346^{a}**	**4.119±0.213^{c}**	**2.338±0.151^{e}**
				α/β-pinene				
**1**	α-pinene	12.14	3.446±0.256^{c}	1.411±0.147^{d}	8.466±0.276^{a}	4.902±0.162^{b}	4.767±0.194^{b}	1.242±0.083^{d}
**3**	β-pinene	14.68	1.133±0.176^{b}	0.257±0.060^{e}	0.661±0.071^{c}	1.951±0.056^{a}	0.551±0.018^{dc}	0.216±0.026^{e}
	**Total**		**4.579±0.4320^{d}**	**1.668±0.207^{e}**	**9.127±0.347^{a}**	**6.853±0.218^{b}**	**5.318±0.212^{c}**	**1.458±0.109^{e}**
				Aliphatic terpenes				
**4**	myrcene	15.90	0.153±0.005^{bc}	0.083±0.004^{d}	0.511±0.049^{a}	0.237±0.022^{b}	0.204±0.006^{b}	0.113±0.012^{cd}
**9**	ocimene	17.97	0.110±0.013^{b}	-	0.173±0.014^{a}	0.008±0.001^{d}	-	0.084±0.011^{c}
	**Total**		**0.263±0.018^{b}**	**0.083±0.004^{d}**	**0.684±0.063^{a}**	**0.245±0.023^{bc}**	**0.204±0.006^{c}**	**0.197±0.023^{c}**
				α/γ/δ-terpinene				
**6**	α-terpinene	16.68	0.081±0.004^{c}	0.151± 0.028^{c}	0.375±0.024^{a}	0.300± 0.008^{ab}	0.300±0.063^{ab}	0.153± 0.016^{c}
**10**	γ-terpinene	18.62	-	0.005±0.001^{c}	0.298±0.065^{b}	0.407±0.009^{a}	0.013±0.000^{cd}	0.020±0.007^{c}
**12**	δ-terpinene	19.75	0.018±0.001^{d}	0.025±0.005^{d}	0.097±0.012^{ab}	0.112±0.003^{a}	0.072±0.009^{c}	0.017±0.004^{d}
	**Total**		**0.099±0.005^{e}**	**0.181±0.034^{d}**	**0.770±0.101^{b}**	**0.819±0.020^{a}**	**0.385±0.072^{c}**	**0.190±0.027^{d}**

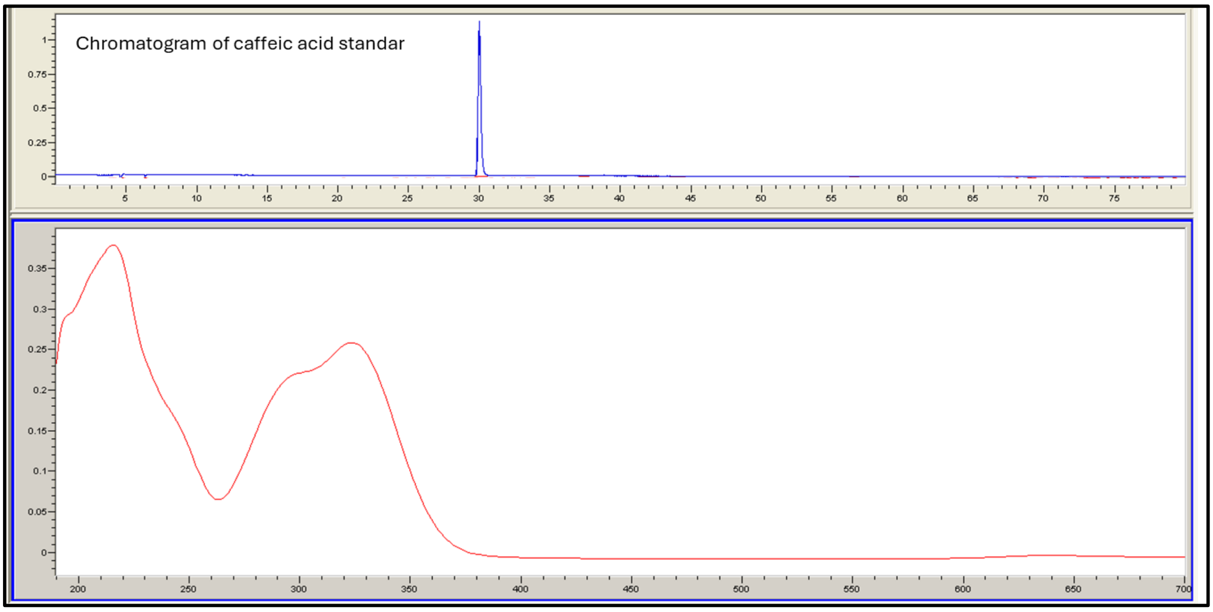

Figure 1. Chromatogram of caffeic acid standard RT= 29.95 min, and UV spectrum. Standard using for the quantification peak as follows: 1.-caffeic acid; 2.-caffeic acid-deriv.

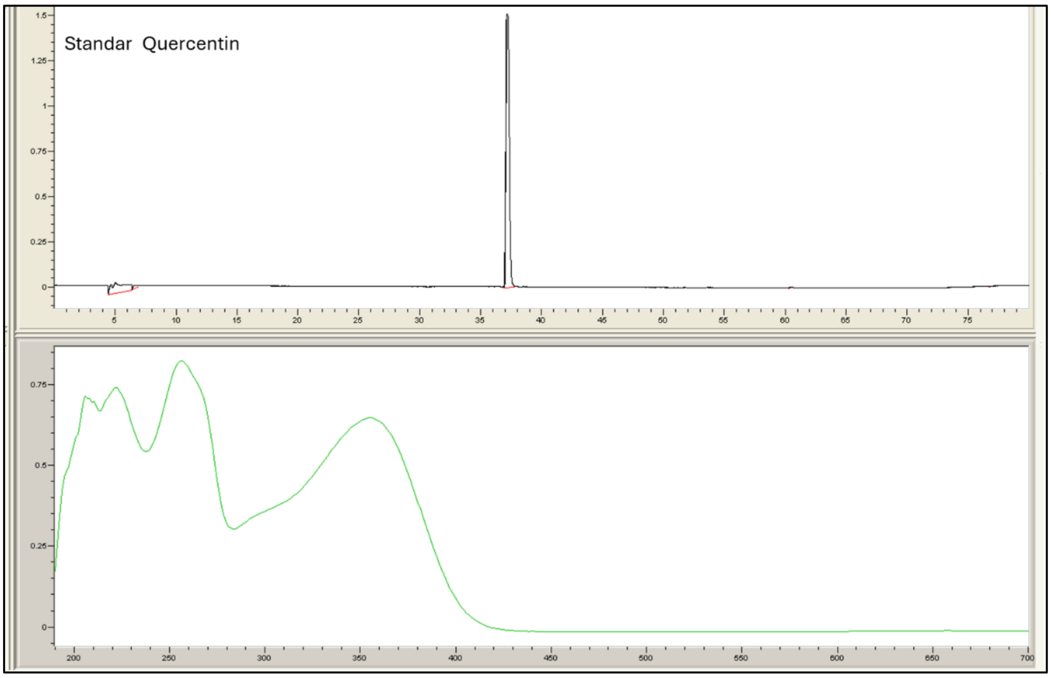

Figure 2. Chromatogram of Quercentin standard RT= 37.42 min, and UV spectrum. Standard using for the quantification peak as follows: 3.-Quecerin deriv-1; 4.- Quecerin deriv-2; 6.- Quecerin deriv-3

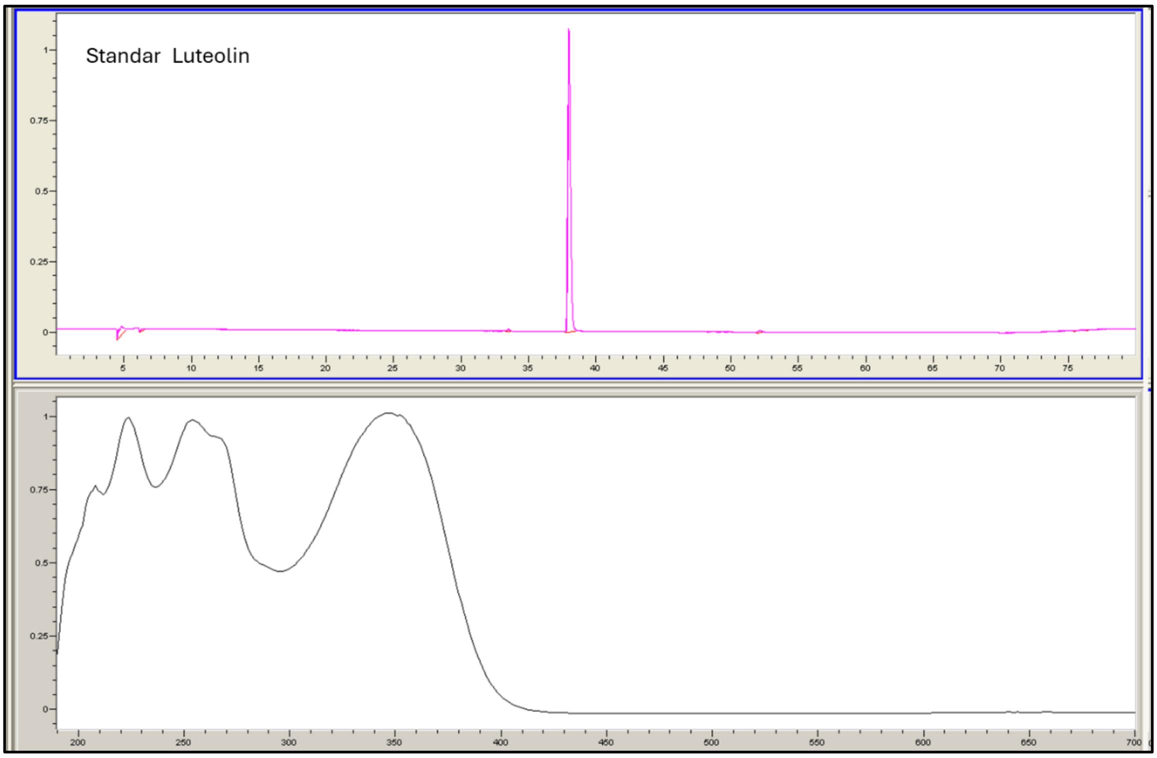

Figure 3. Chromatogram of Luteolin standard RT= 38.81 min, and UV spectrum. Standard using for the quantification peak as follows: 5.-Luteolin-deriv-1; 10.-Luteolin-deriv-2; 12.-Luteolin-deriv-3; 13.-Luteolin-deriv-4; 15.-Luteolin-deriv-5; 16.-Luteolin-deriv-6; 17.-Luteolin-deriv-7.

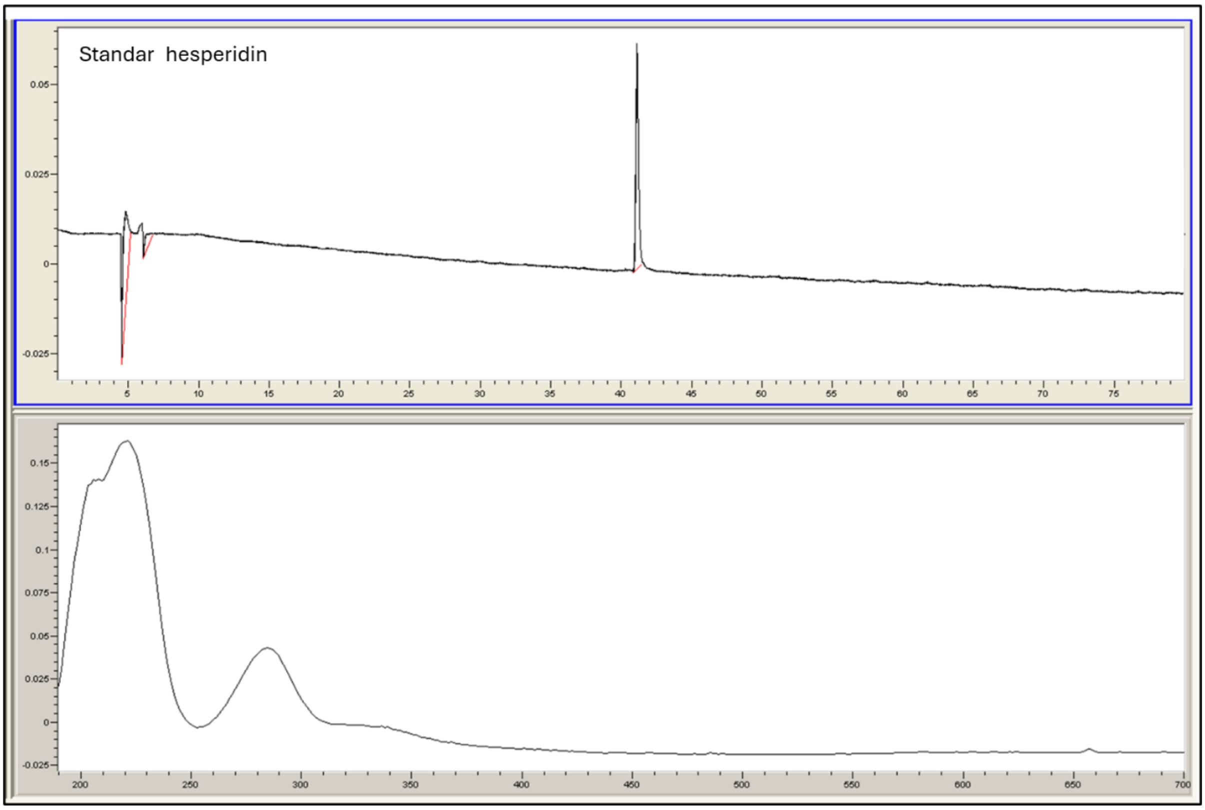

Figure 4. Chromatogram of Hesperidin standard RT= 40.98 min, and UV spectrum.

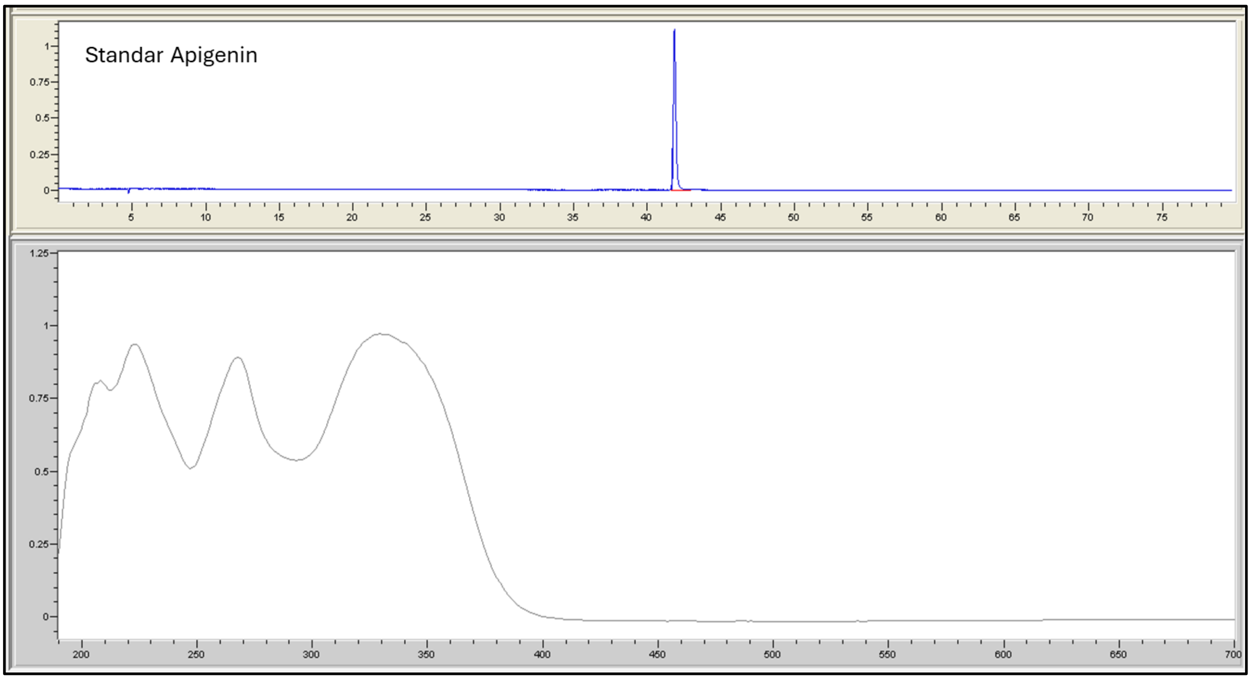

Figure 5. Chromatogram of Apegenin standard RT= 41.89 min, and UV spectrum. Standard using for the quantification peak as follows: 8.-Apigenin-deriv-1; 11.-Apigenin-deriv-2; 14.-Apigenin-deriv-3.

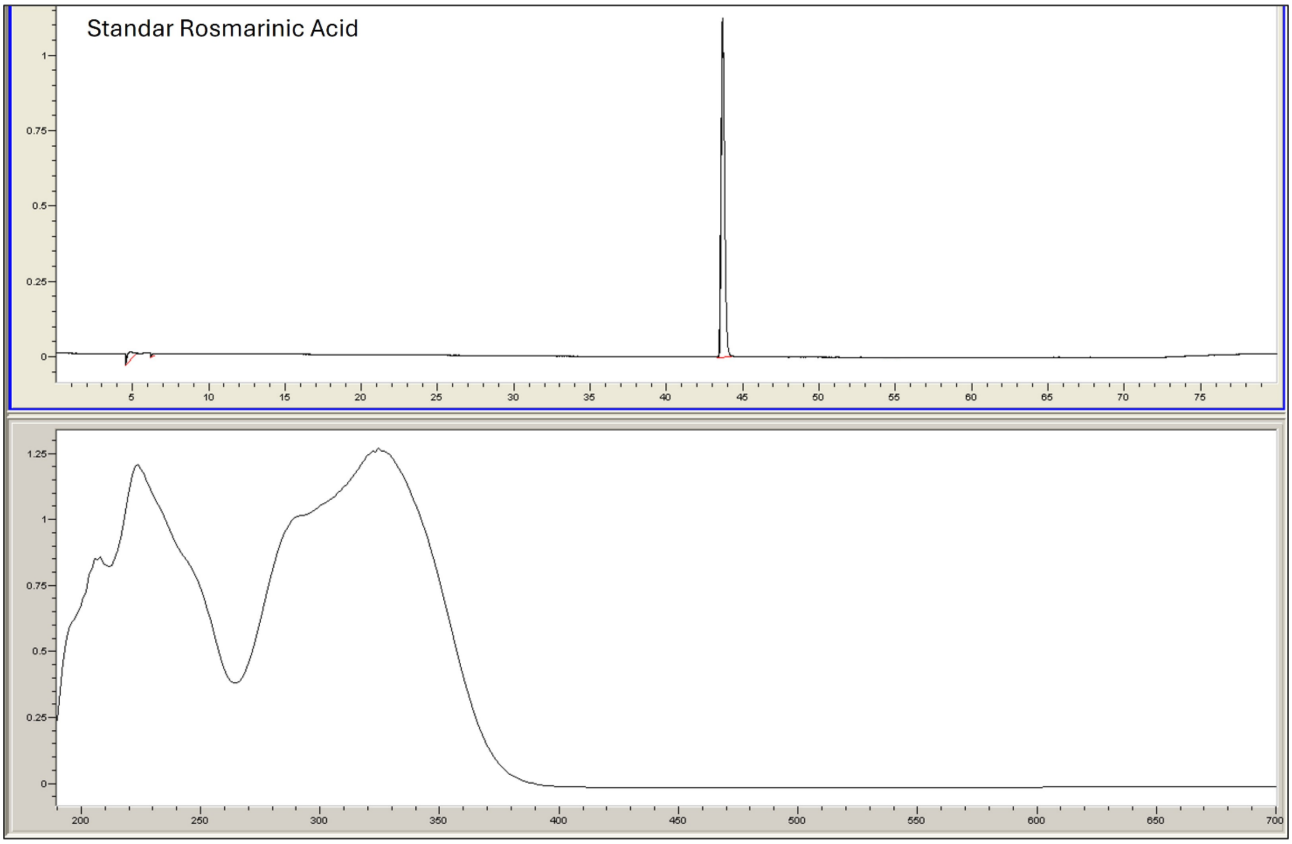

Figure 6. Chromatogram of Rosmarinic acid standard RT= 43.37 min, and UV spectrum. Standard using for the quantification peak as follows: 9.-Rosmarinic acid; 18.-rosmarinic acid deriv-1.

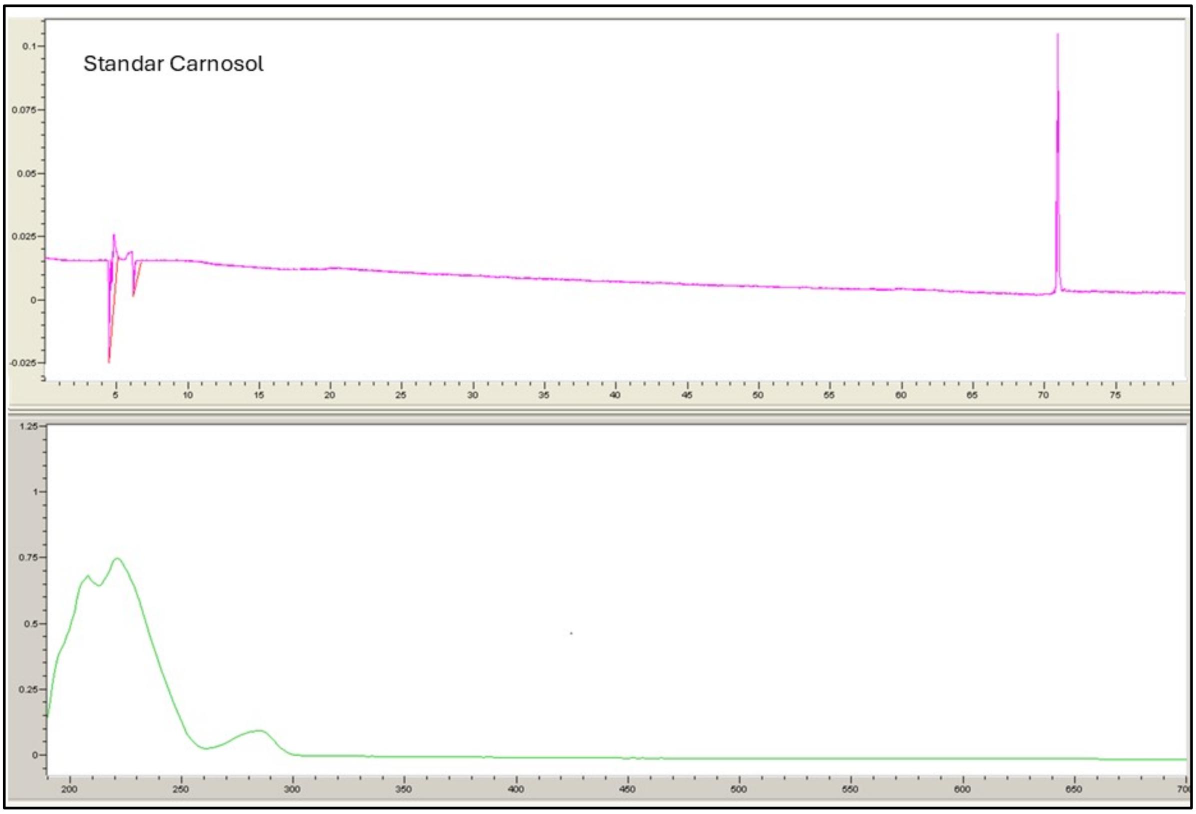

Figure 7. Chromatogram of Carnosol standard RT= 71.11min, and UV spectrum

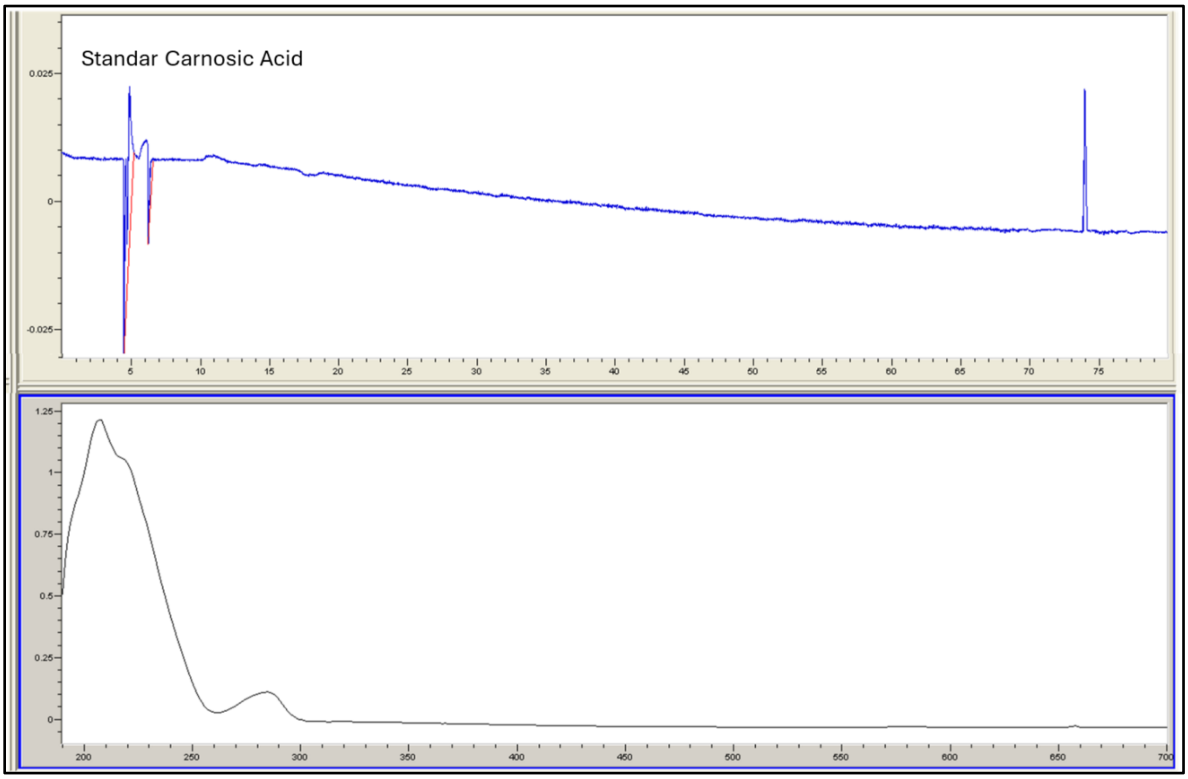

Figure 8. Chromatogram of Carnosic Acid standard RT= 74.46 min, and UV spectrum. Standard using for the quantification peak as follows: 20.-Carnosic acid; 21.-Carnosic acid deriv-1; 22.-Carnosic acid deriv-2.
